# Obturator Foramen Bypass: Long Term Results of a French Case Series

**DOI:** 10.1016/j.ejvsvf.2026.01.001

**Published:** 2026-01-08

**Authors:** Federico Pascucci, Jennifer Canonge, Marie Corniquet, Frédéric Cochennec, Jean Sénémaud, Joseph Touma, Pascal Desgranges

**Affiliations:** a Department of Vascular Surgery, Henri Mondor University Hospital, Créteil, France; b Department of Vascular and Endovascular Surgery, Pitié-Salpêtrière Hospital, Sorbonne Université, Paris, France; c Université Paris Est, Créteil, France

**Keywords:** Aortobifemoral bypass (ABFB), Common femoral artery (CFA), Obturator foramen bypass (OFB), Superficial femoral artery (SFA), Surgical site infections, Vascular infections

## Abstract

**Introduction:**

Obturator foramen bypass (OFB) is an extra-anatomic lower limb revascularisation technique useful in hostile groin cases. Although described during the 1960s, this operation is often considered technically demanding and not widely used nowadays. This study reports the long term outcomes of a series of OFBs.

**Method:**

This was a retrospective, observational, single centre study. All consecutive patients who underwent an OFB between 2002 and 2024 at the Henri Mondor University Hospital were included. Pre-operative patient characteristics, as well as intra-operative and post-operative data were analysed. Primary outcomes were survival and primary and secondary patency. Secondary outcomes were wound healing, freedom from re-infection, and limb salvage.

**Results:**

Between 2002 and 2024, 26 patients underwent an OFB; 18 were men (69%). The mean age was 64 ± 12 years. In 21 cases (81%) the surgical indication was extensive groin infection with native vessels or prosthetic material involvement. In six cases (23%), the operation was performed under emergency conditions. A cryopreserved arterial allograft was the conduit of choice in 16 cases (62%). Mean follow up was 37 ± 8 months (range, 0–129). The thirty day mortality rate was 15%. Survival rate at 12, 24, and 36–60 months was 73%, 67%, and 60%, respectively. The primary patency rate was 88% at 12 months and 83% at 24–60 months. An early re-intervention was needed in eight patients (31%), mostly for local wound debridement. There were no cases of bypass re-infection requiring removal.

**Conclusion:**

Obturator foramen bypass can be a limb and lifesaving procedure in selected cases and experienced centres. Long term patency and wound healing rates are satisfactory. The authors reported a non-negligible early mortality rate, which is associated with the severe initial condition of these patients, often admitted with septic or haemorrhagic shock, and affected by multiple comorbidities. Thorough knowledge of the technique and continuous post-operative follow up are mandatory to eventually prevent and treat complications.

## Highlights

• Even if confined to the femoral arteries, vascular infections have poor outcomes.
• Obturator Foramen Bypass still represents a valid option in hostile groin cases.
• Modern results demonstrate that the technique is reproductible and with good results.
• The early mortality rate is linked to the initial severe status of these patients.
• Long term patency and re-infection rates are very satisfying.

## INTRODUCTION

**Figure 1:**
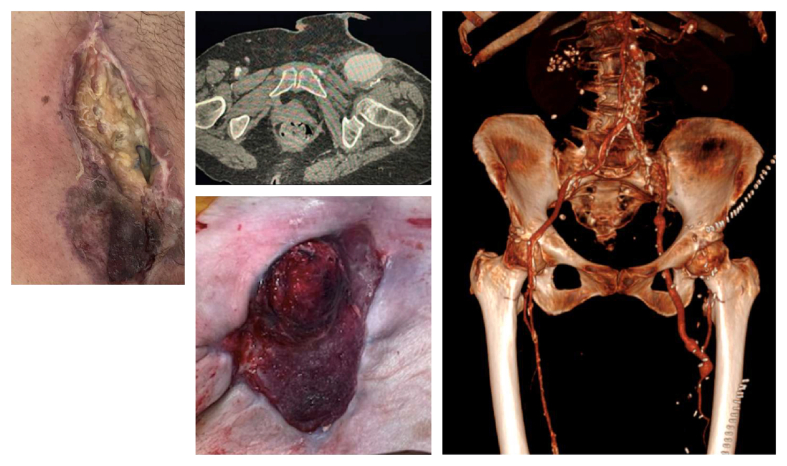
Pre-operative images showing typical clinical cases of femoral prosthetic skin erosion (left) and femoral pseudoaneurysm with cutaneous fistula (middle), treated with OFB. Post-operative 3D computed tomographic angiography reconstruction after a left OFB for an infected femoral pseudoaneurysm (right).

The management of extensive groin infections involving the femoral vessels or prosthetic material remains a critical issue in vascular surgery, with non-negligible morbidity and mortality rates despite optimal care.^1^ Obturator foramen bypass (OFB) is one of the available options that permits lower limb revascularisation, reducing the risk of post-operative bypass exposure or re-infection.^2^ Initially described by Shaw and Baue^3^ at the beginning of the 1960s, the OFB is an extra-anatomic iliofemoral reconstruction that restores perfusion to the lower limb via theoretically clean tissue planes, circumventing infected groin areas.^3^^,^^4^ The original technique provides a standard intra- or extraperitoneal approach to the aorta or iliac arteries to obtain arterial inflow and usually a medial approach to the superficial or deep femoral artery. The tunnel is made through the obturator membrane in its medial part, avoiding the obturator vessels and nerve, continuing in the thigh beneath the adductor longus muscle to rejoin the chosen outflow artery. Over the decades, its application has been extended to aortobifemoral bypass (ABFB) infections, complex femoral lesions, such as infected pseudoaneurysms in drug users and hostiles groins following radiation or multiples operations (Fig. 1).^5^^,^^6^

Despite encouraging results, and various attempts to simplify the technique, the OFB did not gain the expected uptake by the vascular community, probably for its technical complexity.^7^ Recent series have reinforced its role in vascular surgery showing very satisfying results. A French single centre study involving 22 patients demonstrated primary patency rates of 84%, 78%, and 63% at one, two, and three years, respectively. Infection control was obtained in 100% of patients, and wound healing was achieved in 90% of cases.^8^ Bath *et al.*^9^ recently published a series of 18 OFBs performed with ePTFE conduits. The authors reported only one case of re-infection requiring removal at follow up. The primary patency rate was 65% at 24 months, while primary assisted and secondary patency rates were 71% and 88% at 24 months, respectively. At 36 months the survival rate was 83%, while three patients underwent a major amputation.^9^ Masaki *et al.*^10^ reported a series of 16 patients, with a three and five year primary patency rate of 69% and 43%, respectively. The survival rates were 69% and 59% at three and five years and no patient reported bypass re-infection. Other small OFB series reported very different patency and survival rates, often lacking in long term follow up. This study reports long term outcomes of a series of 26 consecutive OFB cases performed at a single centre over a 22 year period.

## METHOD

### Study design

**Figure 2:**
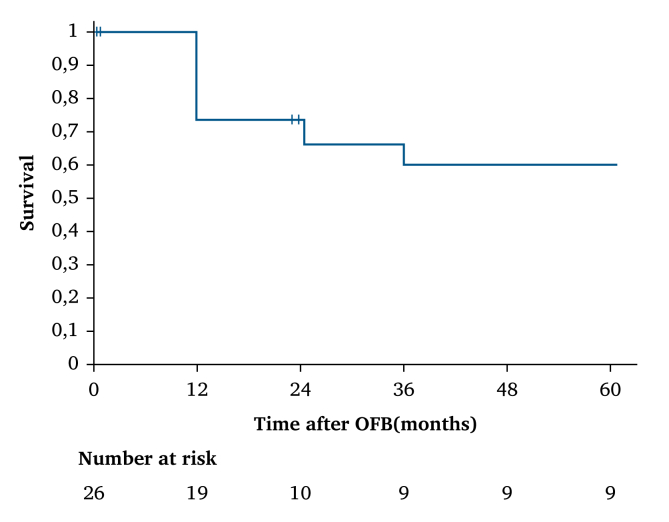
Kaplan–Meier analysis reporting five year survival estimations.

All patients who underwent an OFB at the Henri Mondor University Hospital between 2002 and 2024 were included in this study. Given the nature of the study, institutional review board approval was waived. This case series was reported in accordance with the PROCESS guidelines. Pre-operative patient characteristics and surgical history, in hospital and operative reports, as well as post-operative outcomes and additional hospitalisations were collected prospectively and analysed retrospectively. Clinical follow up was usually performed post-operatively at one, three, and six months, and yearly thereafter, unless more frequent follow up was considered necessary by the surgeon. Post-operative groin wound healing was achieved with vacuum assisted closure (VAC) therapy or a simple secondary healing process, depending on local conditions. Regarding the operative technique, OBF was performed in a standard fashion following the previously described technique (Fig. 2).^7^ Access to native aorto-iliac arteries or to the limb of a pre-existing ABFB was obtained through a standard intra- or extraperitoneal approach, depending on the operating surgeon's preference. Inflow and outflow arteries were chosen depending on pre-operative imaging, surgical history, and local conditions. The superficial or deep femoral artery was dissected through a standard medial approach. The tunnelling pathway was carefully developed through the obturator membrane, avoiding the obturator vessels and nerve, and thereafter beneath the adductor longus muscle.

### Statistical analysis

Data were analysed with SPSS 17 for Windows (SPSS, Inc, Chicago, IL, USA). Quantitative variables were described using the mean ± standard deviation (SD), while qualitative variables were summarised using frequencies and percentages. The Kaplan–Meier method was used to estimate survival and patency rates. All analyses were purely descriptive. Quantitative variables were reported as mean ± SD. Qualitative variables were presented as absolute frequencies and percentages. No inferential statistics were performed.

## RESULTS

### Demographic results

Twenty-six patients underwent an OFB during the 22 year study period. The authors did not report any bilateral OFB cases. Demographics, comorbidities, and surgical indication are summarised in Table 1. The mean age was 64 ± 12 years, and 18 patients (69%) were men. All patients had a history of previous vascular surgery. Previous aortic surgery was reported in nine patients (35%), previous iliac revascularisation in 12 (46%) and previous femoropopliteal revascularisation in 17 patients (65%). Surgical indication was an extensive groin infection in 21 cases (81%), a femoral pseudoaneurysm in three cases (11%) and a hostile groin for previous multiple operations in two cases (8%). In six cases (23%), the operation was performed under emergency conditions due to haemorrhagic shock. A cryopreserved arterial allograft was the conduit of choice in 16 cases (62%). In four patients (15%) an autologous great saphenous vein was used, while in three patients (11%) superficial femoral veins were used (Table 2). Arterial inflow was obtained from the abdominal aorta in six patients (23%) and from iliac arteries in 17 patients (65%). In three cases (12%) the limb of a previous ABFB served as arterial inflow (Table 2) (see Table 3).

**Table 1:** Patients’ characteristics.

Variable	Absolute number (%) or mean ± SD (total 26 patients)
Demographics	
*Age – y*	63 ±12
*Male sex*	18 (69)
Comorbidities	
*Current or past smoker*	18 (69)
*Hypertension*	21 (80)
*Diabetes*	8 (30)
*Hyperlipidaemia*	17 (65)
*Obesity*	8 (31)
*Chronic kidney disease*	1 (4)
*Coronary artery disease*	11 (42)
*Atrial fibrillation*	2 (8)
*Previous cancer history*	3 (12)
Previous surgical history	
*Previous aortic surgery*	9 (35)
*Previous iliac revascularisation*	12 (46)
*Previous femoropopliteal revascularisation*	17 (65)
*Previous major amputation*	4 (15)

**Table 2:** Peri-operative data.

Variable	Absolute number (%) (total = 26 patients)
Indication for obturator foramen bypass	
*Scarpa's triangle infection*	21 (81)
*Femoral pseudoaneurysm*	3 (11)
*Multiple previous Scarpa's triangle operations*	2 (8)
Material chosen for bypass	
*Cryopreserved arterial allograft*	16 (62)
*Great saphenous vein*	4 (15)
*Superficial femoral vein*	3 (11)
*Prosthetic material*	2 (8)
*Mixed materials*	1 (4)
Arterial inflow	
*Aorta*	6 (23)
*Iliac artery*	17 (65)
*Previous aortobifemoral bypass*	3 (12)

**Table 3:** Detailed description of surgical cases.

Case	Demographics (age – y, sex)	Surgical indication for OFB	Operative management	Material used for OFB	30 d death	Early re-intervention
1	66, M	Iliofemoral bypass infection with cutaneous fistula	Infected bypass removal + iliofemoral OFB	Arterial allograft	No	No
2	71, F	Femoropopliteal bypass infection after percutaneous fibrinolysis for acute limb ischaemia	Infected bypass removal + iliofemoral OFB	GSV	No	Yes
3	60, M	Groin infection with patch pseudoaneurysm after previous femoral endarterectomy	Iliofemoral OFB + sartorius rotational flap coverage	GSV	No	No
4	62, M	Groin infection after previous axillobifemoral bypass	Infected bypass removal + Aortofemoral OFB + bilateral sartorius rotational flap coverage	Arterial allograft	No	No
5	67, F	Septic distal anastomotic rupture of redo ABFB made in arterial allograft for previous prosthetic ABFB infection	Partial allograft removal and iliofemoral OFB	Arterial allograft	No	No
6	66, F	Femorofemoral crossover bypass infection made for a previous ABFB limb occlusion	Iliofemoral OFB + femoropopliteal bypass + contralateral axillofemoral bypass	Arterial allograft, SFV, SFA	No	Yes
7	50, M	Prosthetic ABFB infection	Infected bypass removal + new ABFB in arterial allograft via obturator foramen	Arterial allograft	No	No
8	64, F	Infected groin haematoma after previous transfemoral cerebral aneurysm embolisation	infected haematoma evacuation + iliofemoral OFB	Arterial allograft	No	No
9	68, M	Iliofemoral prosthetic bypass infection	Infected bypass removal + aortofemoral OFB	Arterial allograft	No	No
10	52, M	ABFB and femoropopliteal bypass infection	Infected bypass removal and new ABFB through obturator foramen + new femoropopliteal bypass	Arterial allograft	No	No
11	71, M	Severe acute limb ischaemia on iliofemoral occlusion after femoral endarterectomy complicated by SSI treated by sartorius rotational flap coverage	Aortofemoral OFB	ePTFE prosthesis	No	No
12	54, F	ABFB and femorofemoral crossover bypass infection	Infected bypass removal and new ABFB through obturator foramen + selective bypasses to SMA and renal arteries from thoracic aorta	Arterial allograft	Yes	No
13	55, M	Iliofemoral bypass occlusion and groin infection	Aortofemoral OFB + femoropopliteal bypass	Arterial allograft	No	No
14	58, M	Septic common femoral artery rupture after ECMO	Iliofemoral OFB + sartorius rotational flap coverage	GSV	No	No
15	74, M	Septic femoral pseudoaneurysm after previous PCI	Pseudoaneurysm excision + Iliofemoral OFB	Arterial allograft	Yes	No
16	55, M	Mycotic external iliac artery aneurysm	Mycotic aneurysm excision + Iliofemoral OFB	Arterial allograft	No	No
17	64, M	CLTI on multiples previous groin operations	Iliofemoral OFB	SFV	No	No
18	56, M	Severe acute limb ischaemia on multiples previous iliofemoral bypasses	Iliopopliteal OFB	GSV	No	No
19	72, M	Groin infection on previous ABFB and femoropopliteal prosthetic bypass	Infected partial bypass removal + prosthetic - femoral OFB	Arterial allograft	No	No
20	78, M	Groin infection on previous femoropopliteal prosthetic bypass	Infected bypass removal + Iliofemoral OFB	SFV	Yes	Yes
21	63, M	Groin infection on previous aortofemoral and femoropopliteal prosthetic bypass	Infected bypass partial removal + Prosthetic - femoral OFB	Arterial allograft	Yes	Yes
22	63, M	Anastomotic femoral pseudoaneurysm on previous ABFB	Prosthetic - femoral OFB	ePTFE prosthesis	No	Yes
23	71, F	Septic femoral pseudoaneurysm after previous cerebral aneurysm embolisation	Pseudoaneurysm excision + Iliofemoral OFB	Arterial allograft	No	Yes
24	77, F	Iliofemoral and femoropopliteal prosthetic bypass infection	Infected bypass removal + iliofemoral OFB	Arterial allograft	No	Yes
25	79, F	Septic ruptured pseudoaneurysm on previous iliofemoral bypass	Infected bypass removal + Iliofemoral OFB	SFV	No	No
26	49, M	Groin incision infection with Aortopopliteal prosthetic bypass macroscopic exposition	Infected bypass partial removal + Iliopopliteal OFB	Arterial allograft	No	Yes

M = male; F = female; ABFB = aortobifemoral bypass; OFB = obturator foramen bypass; GSV = great saphenous vein; SFV = superficial femoral vein; SFA = superficial femoral artery; ePTFE = expanded polytetrafluoroethylene; SSI = surgical site infection; ECMO = extracorporeal membrane oxygenation; PCI = percutaneous coronary intervention; CTLI = chronic limb threatening ischaemia; SMA = superior mesenteric artery.

### Operative outcomes and long term results

**Figure 3:**
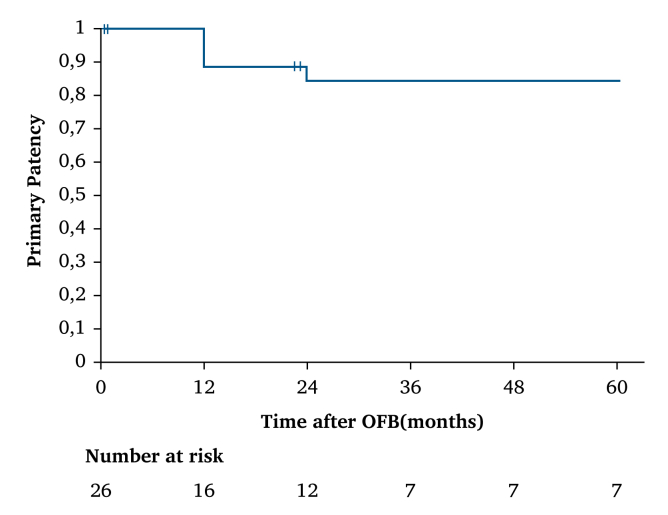
Kaplan–Meier analysis reporting five year primary patency estimations.

There were no intra-operative deaths. The mean follow up was 37 ± 8 months (range, 0–129). Four patients died in the first 30 post-operative days (15%). Survival rates at 12, 24, and 36–60 months were 73%, 67%, and 60%, respectively (Fig. 3). The primary patency rate was 88% at 12 months and remained stable at 83% from 24–60 months. Graft thrombosis occurred only in one case in the early post-operative period due to insufficient runoff. Surgical treatment consisted of thrombectomy and adjunctive distal bypass. In two cases, after three and six months, a distal bypass was necessary due to insufficient lower limb vascularisation. A distal anastomotic angioplasty was necessary in only one case after 15 months to prevent graft thrombosis. There were no OFB re-infections requiring removal. Limb salvage was achieved in all but one case, in which an early transfemoral amputation was needed for a stage VI Rutherford chronic limb threatening ischaemia patient with sepsis. Complete wound healing was achieved in all patients with a median delay of six months using either VAC therapy or simple secondary healing. In eight patients (31%) early re-intervention was necessary in the first 30 post-operative days, in half of the cases only for local wound debridement or local haemostasis. Other re-interventions were a covered stenting for a distal anastomotic pseudoaneurysm and the abovementioned transfemoral amputation and thrombectomy and distal bypass for early OFB occlusion.

## DISCUSSION

Surgical management of vascular infections has historically been based on total removal of infected materials, aggressive debridement of surrounding tissues and extra-anatomic or *in situ* vascular reconstruction. *In situ* reconstruction seems to provide better results in the surgical treatment of aortic graft and endograft infections, especially avoiding the catastrophic eventuality of aortic stump blowout.^11^ Anastomosis and implanted material coverage with omentum, muscle flaps, or biological patches also is recommended in these cases.^2^ Extra-anatomic revascularisations, however, still maintain a fundamental role in lower limbs, avoiding new bypass passing through an infected field. Even materials considered highly resistant to re-infections, such as cryopreserved arterial allografts, showed great frailties when used in situ for peripheral vascular graft infections. Analysing a series of 53 patients treated with in situ cryopreserved arterial allografts, the graft related re-intervention rate was 33% at five years, mostly for allograft related complications such as rupture, aneurysmal degeneration, or anastomotic pseudoaneurysm.^12^

To date, despite remarkable improvements in surgical techniques, the optimal material for vascular reconstructions in infected fields has yet to be identified. In such cases, biological materials such as autologous veins or cryopreserved arterial allografts, have shown excellent patency rates and resistance to re-infection. The use of femoropopliteal veins has progressively gained interest in aortic and peripheral reconstructions for native or prosthetic infections, despite more complex procedures and longer operation times.^13^^,^^14^ At the same time, when available, cryopreserved arterial allografts share the same advantages in terms of resistance to re-infection, avoiding extensive incisions for vein harvesting, with similar results in aortic and peripheral procedures globally.^15^^,^^16^ In recent years, the authors have also observed an increase in use of tubulised xenopericardial substitutes, with satisfying results in aorto-iliac and infrainguinal reconstructions.^17^ An alternative off the shelf biological substitute often used in these settings is the hybrid synthetic biological prosthesis known as the Omniflow graft (Lemaitre Vascular, Burlington, MA, USA). The Omniflow graft is composed of a polyester mesh endoskeleton covered by cross linked ovine collagen. Reports of its use in septic settings remain limited but early results suggest this graft may offer greater resistance to infection than rifampicin soaked or silver coated grafts.^18^ Conversely, when radical surgery was not considered indicated or feasible, various combinations of conservative techniques have been published such as sartorius or gracilis muscle flap coverage, associated or not with adjunctive VAC therapy.^19^ In such cases, Dua *et al.*^20^ reported an excellent healing rate of 100% at three months with adjunctive VAC therapy and lifelong antibiotics. However, only 60% of patients were alive at 24 months, with only 33% of grafts still patent at that time.^20^ Considering these data, in the authors’ practice, conservative treatment is reserved only for patients considered unfit for surgery.

When treating local septic conditions, the theoretical advantages of extra-anatomic bypasses, assuring efficient revascularisations via theoretically clean planes, circumventing infected areas, appear evident. Another interesting but rarely performed extra-anatomic lower limb revascularisation is the transiliac wing bypass. Initially described by Brzezinski *et al.*^21^ in 1989, this bypass is particularly useful in case of a hostile pelvis for surgery or radiation, but also for extensive groin infection.^21^, ^22^, ^23^, ^24^ However, a potential disadvantage of this technique is the anatomic disposition and route of the bypass itself, which is particularly prone to severe kinking, increasing the risk of thrombosis. At the same time, the increased bypass length may be a thrombosis risk factor. Some authors also evoked the potential risk of severe bleeding caused by a transiliac wing tunnel.^25^ For these reasons, if not contraindicated for a medical history of pelvic surgery of radiation, OBF remains the first choice in surgical practice when needed. Despite advances in surgical techniques and peri-operative management, these results confirm that surgical treatment of vascular infections, if confined to femoral vessels, still poses a heavy burden on short term outcomes. In this study population, the authors observed a non-negligible rate of early death, mostly due to the severe initial clinical condition of these frail patients, often operated on under emergency conditions for sepsis or haemorrhagic shock, and to the magnitude of a long and haemorrhagic operation. At the same time, frequent early re-interventions demonstrate the need for careful post-operative monitoring to detect early complications. Tunnel creation, historically considered technically demanding and a potential source of important bleeding due to damage to the obturator vessels, has probably been the cause of rare OFB uptake by the vascular surgery community. Despite this, the authors consider this technique feasible and reproductible, and they strongly encourage its demystification, particularly for younger surgeons. On the other hand, the satisfying long term patency rate of OFB in this series seems to be an important positive characteristic of this revascularisation that needs to be considered. As the mean age of patients in this series was relatively young, considerations on the operation's durability needs to be carefully weighted.

In conclusion, OFB still represents a valuable lower limb revascularisation option in hostiles groin cases for infections, pseudoaneurysms, or multiple operations. Contemporary data show excellent long term patency rates, infection resolution and limb salvage, making this technique a valuable permanent iliofemoral revascularisation. It must be noted that the procedure itself carries a non-negligible early mortality rate, mainly due to the patient's critical condition on admission. Although it requires advanced surgical skills and regular follow up, OFB should be demystified in the vascular surgery community. Considering modern results, the technique deserves consideration as a durable extra-anatomic revascularisation strategy in complex aorto-iliofemoral reconstructions, especially in infected fields.

## Funding

This research did not receive any specific grant from funding agencies in the public, commercial, or not-for-profit sectors.
